# A mini-review on the most important effective medicinal plants to treat hypertension in ethnobotanical evidence of Iran

**Published:** 2016-06-14

**Authors:** Babak Baharvand-Ahmadi, Majid Asadi-Samani

**Affiliations:** ^1^Madani Heart Hospital, Lorestan University of Medical Sciences, Khorramabad, Iran; ^2^Student Research Committee, Medical Plants Research Center, Shahrekord University of Medical Sciences, Shahrekord, Iran

**Keywords:** Ethnobotany, Medicinal plants, Hypertension

## Abstract

Nowadays, cardiovascular diseases are highly prevalent in human communities. Hypertension is a multifactorial disease which causes a mortality twice higher than general population. Given the fact that medicinal plants have long been used to treat hypertension and are currently being administered for this disease, we sought to report the mostly effective and important medicinal plants on hypertension therapy in ethno-botanical evidence of Iran. In this study, hypertension, Iran, ethno-botany, medicinal plants, and traditional medicine were used as key words to search in Web of Science, PubMed, Scopus, EBSCO and EMBASE to select relevant articles. The findings of this study indicated that in Iran 40 plants in various provinces are used to treat hypertension. Because medicinal plants in this study contain effective compounds and have long been used to treat and reduce hypertension, they could provide suitable research arrangements for controlling hypertension, while effective natural drugs could be developed to control hypertension if their properties are confirmed in pharmacological studies.

**Implication for health policy/practice/research/medical education:**

The medicinal plants may prevent or treat various diseases through exerting antioxidant and anti-inflammatory properties. Since they have long been applied to treat and reduce hypertension, they can be used to develop effective and natural drugs to control high blood pressure, however, their effect should confirm by pharmacological investigations and clinical trials.

## Introduction

Ethno-botany or traditional botany deals with the traditional therapeutic properties of the medicinal plants used by people of different cultures and regions (1-3). Since our surrounding nature and the flora of various regions is full of plant species with unknown pharmaceutical and therapeutic properties and discovering their properties may take a long time, then ethno-botany is useful because of transforming the pharmaceutical culture and herbal medicine of any regions from oral into a written one (4-7). Traditional knowledge and administration of medicinal plants help pharmaceutical industry develop new drugs and to use them for prevention and treatment of various diseases (8-10).

The people of different cultures and nations worldwide, particularly in Iran, China, Egypt, and Greece, have been assumed, chemical drugs are efficient to improve disease and expedite this process, however, medicine has been advancing and any chemical drugs, alongside therapeutic properties, have been demonstrated to cause side effects, the people have opted to use the medicinal herbs with minimal side effects (11-19). In this regard, different studies have investigated the effect of medicinal herbs for prevention and treatment of neurological disorders and infectious, gastrointestinal, and respiratory diseases. Beyond the effects of medicinal plants on neurological disorders, wounds, a variety of pains, migraine, cold, diabetes, hypertension, hyperlipidemia, skin problems, peptic ulcer, dysmenorrhea, and reproductive system have been studied (20-41).

Nowadays, cardiovascular diseases are highly prevalent in human communities and their treatment is a health priority in many countries. Among cardiovascular diseases, hypertension is a serious cardiovascular disease and mortality rate in the patients with hypertension is twice higher than general population. Hypertension contributes significantly to increasing sudden death in cardiac diseases patients, intensified by risk factors such as smoking, diabetes, and hypercholesterolemia (42).

Since medicinal plants have long been administered and are still being used to treat hypertension, and some of chemical drugs fail to meet patients all needs and some of them may lead to side effects, hence this review article aimed to study the medicinal plants efficacy on hypertension in ethno-botanical evidence of Iran. This study secondly aimed to provide pharmacists and researchers with some ideas on development of the medicinal plants for hypertension therapy.

## Materials and Methods

For this review, we used a variety of sources by searching through Web of Science, PubMed, EMBASE, Scopus and directory of open access journals (DOAJ). The search was performed by using combinations of the following key words and or their equivalents; hypertension, Iran, ethno-botany, medicinal plants, and traditional medicine.The articles with non-English full text were excluded from this review article.

## Results

The findings of this study indicated that 40 medicinal plants are used in different provinces of Iran for treatment of hypertension. These plants are mostly from Asteraceae, Polygonaceae, Rosaceae, and Oleaceae families. Most plants have grown in different regions of Zagros mountains. The native plants of Iran that are effective on hypertension are listed in Table 1.

**Table 1:** Effective medicinal plants on hypertension by ethno-botanical evidence of Iran

**Row**	**Scientific name**	**Family**	**Persian name**	**Part of plants**	**Distinct**
1	*Allium sativum L.*	Aliaceae	Sir	Root	West Azerbaijan (43)
2	*Juglans regia L.*	Juglandaceae	Gerdou	Leaves and fruit	West Azerbaijan (43)
3	*Berberis vulgaris* L.	Berberidaceae	Zereshk	Leaves and fruit	Arasbaran (44)
4	*Achillea millefolium* L.	Asteraceae	Boumadaran	Shoot	Arasbaran (44)
5	Ecbalium elaterium	Cucurbitaceae	Khiare vahshi	Root and fruit	Arasbaran (44)
6	*Ribes orientale*	Grossulariaceae	Angour sharghi	Fruit	Arasbaran (44)
7	*Crataegus monogyna*	Rosaceae	zalzalak	Leaves and fruit	Arasbaran (44)
8	*Crataegus pontica* C. Koch*.*	Rosaceae	zalzalak	Leaves and fruit	Ilam (45)
9	*Paliurus spina-christi* Miller.	Rhamnaceae	Siah tale	Fruit	Ilam (45)
10	*Rheum ribes* L.	Polygonaceae	Rivas	Stem	Ilam (45)
11	*Suaeda altissima*	Chenopodiaceae	A type of Siah shor	Leaves and stem	North East Persian Gulf (46)
12	Olea europea	Oleaceae	Zeytoun	Fruit	North East Persian Gulf (46)
13	*Silybum marianum* L. Gaertn.	Asteraceae	Khar maryam	Stem and root	Khuzestan (47)
14	*Tragopogon aureus* Boiss.	Asteraceae	A type of Sheng	Leaves and fruit	Khuzestan (47)
15	Olea europea	Oleaceae	Zeytoun	Leaves and fruit	Khuzestan (47)
16	*Securigera securidaca* Degen & Dorfl.	Papilionacea	Adas talkh	Seed	Khuzestan (47)
17	*Rumex pulcher* L.	Polygonaceae	Torshak	Root	Khuzestan (47)
18	*Nigella sativa* L.	Ranunculaceae	Siah daneh	Seed	Sistan (48)
19	*Anthemis cotula* L.	Asteraceae	Babouneye bahari	Flower	North Iran (49)
20	*Suaeda altissima* Pall.	Chenopodiaceae	Zeytoun	Leaves and stem	North Iran (49)
21	*Olea europaea* L.	Oleaceae	Zeytoun	Fruit	North Iran (49)
22	*Silybum marianum* (L.) Gaerth.	Asteraceae	Khar maryam	Flower	Kazeroon (50)
23	*Rumex crispus* L.	Polygonaceae	Torshak	Leaves	Mobarakeh (50)
24	*Ziziphus jujuba*(L) H.Karst	Rhamnaceae	Anab	Fruit	Mobarakeh (50)
25	*Olea europaea* L	Oleaceae	Zeytoun	Fruit	Mobarakeh (50)
26	*Echium amoenum* L*.*	Boraginaceae	Gav zaban	Flower	Mobarakeh (50)
27	*Nasturtium officinale* R. Br.	Brassicaceae	Alafe cheshme	Shoot	Marivan (51)
28	*Fumaria asepala* Boiss	Fumariaceaea	Shahtareh bikasbarg	Shoot	Marivan (51)
29	*Rumex conglomerates* Murr	Polygonaceae	Torshak	Leaves and stem	Natanz (52)
30	*Nectaroscordeum tripedale*	Amaryllidaceae	Piaze tabestaneh lorestani	Shoot	Lorestan (53)
31	*Nectaroscordeum coelz*i	Amaryllidaceae	Piaze tabestaneh lorestani	Shoot	Lorestan (53)
32	*Falcaria vulgaris*	Apiaceae	Ghazyaghi	Leaves, flowers and stem	Lorestan (53)
33	*Smyrnium cordifolium*	Apiaceae	Andol	Seed	Lorestan (53)
34	*Crocus hasskenechtii*	Iridaceae	Pishouk	Root	Lorestan (53)
35	*Berberis integrima*	Berberidaceae	Zereshk	Leaves and stem	Lorestan (53)
36	*Ziziphus spina-christi*	Rhamnaceae	Sedr	Leaves, flowers and fruit	Lorestan (53)
37	Ziziphus nummularia	Liliaceae	Konar	Bulb	Lorestan (53)
38	*Allium ursinum*	Asteraceae	Valak	Shoot	Lorestan (53)
39	*Anethum graveolens*	Apiaceae	Shevid	Shoot	Lorestan (53)
40	*Amygdalus scoparia*	Rosaceae	Badam	Fruit	Lorestan (53)

## Discussion

Based on the written evidence originated from thousands of years ago, administration of medicinal plants has been one of the most primitive methods applied by humans to treat various diseases. In addition, documented data from thousands of years ago in pharmacy and medical history imply existence of valuable data on phytotherapy. Additionally, growing interest of researchers of different fields has caused to name the current century as the century of return to nature, and most researchers of pharmacognosy and related sciences have conducted some studies to identify effective substances, pharmacologic property, and therapeutic uses and develop plant-derived drugs to treat a variety of diseases such as cardiovascular and gastrointestinal and cancers, and even to control infertility (54-58). In this regard, the present study intended to investigate the most important medicinal plants that are effective on hypertension in ethno-botanical evidence of Iran, indicated that 40 medicinal plants in different provinces of Iran are administered to treat hypertension, which could provide a good background to do further studies on hypertension control.

Most identified medicinal plants in this study are from Asteraceae, Polygonaceae, Rosaceae, and Oleaceae families. Phytochemical studies have found that many of these plants contain flavonoids and terpenes components flavonoids are one of the most important phenol groups in nature which are abundantly found in Asteraceae and Polygonaceae families and also have been reported to exist in Rosaceae and Oleaceae families. Polyphenols have been known as a protective factor against many diseases such as cardiovascular and a preventive factor for hypertension (59-64). Sesquiterpene lactones are from terpenes family and a salient feature of Asteraceae family. Furthermore, they may be found in certain families including Apiaceae. Over 6000 compounds related to sesquiterpenes have been so far identified in the plants from these families. A feature of all the sesquiterpene lactones which seem to be closely associated with their biological activity is removal of unsaturated gamma-lactone at alpha-beta. Most sesquiterpene lactones are non-toxic and taste bitter. Moreover, most of them have been reported to exert antitumor, antibacterial, cardiotonic and anti-inflammatory effects and relax smooth muscles (65-69). Polystyrenes are another large group of secondary metabolites. To date, over 1400 polystyrenes and the compositions derived from them have been isolated and identified. Polystyrenes are commonly found in the plants from Araliaceae, Apiaceae, and Asteraceae families. Polystyrenes exert considerable toxic side effects against fungi, bacteria, breast carcinoma cells. These compounds also exert anti-platelet aggregation properties (70).

## Conclusion

The medicinal plants may prevent or treat various diseases through exerting antioxidant and anti-inflammatory properties. Since they have long been applied to treat and reduce hypertension, they can be used to develop effective and natural drugs to control high blood pressure, however, their effect should confirm by pharmacological investigations and clinical trials.

## Authors’ contribution

BBA completed the article and MAS reviewed the article. All authors read and signed the final draft.

## Conflicts of interest

The authors declared no competing interests.

## Ethical considerations

Ethical issues (including plagiarism, data fabrication, double publication) have been completely observed by the authors.

## Funding/Support

None.
